# Movement Asymmetries: From Their Molecular Origin to the Analysis of Movement Asymmetries in Athletes

**DOI:** 10.3390/life13112127

**Published:** 2023-10-27

**Authors:** Alexander Egoyan, Giorgi Parulava, Steven Baker, Melinda Gilhen-Baker, Giovanni N. Roviello

**Affiliations:** 1 Faculty of Physical Medicine and Rehabilitation, Georgian State Teaching University of Physical Education and Sport, 49 Chavchavadze Avenue, 0162 Tbilisi, Georgia; alexander.egoyan@sportuni.ge (A.E.); giorgi.parulava@sportuni.ge (G.P.); mgbaker@me.com (M.G.-B.); 2 Compete Physiotherapy Ltd., Unit 1, Bridge Mill, Cowan Bridge, Carnforth LA6 2HS, UK; steve@competephysio.co.uk; 3 Institute of Biostructures and Bioimaging, Italian National Council for Research (IBB-CNR), Area di Ricerca Site and Headquarters, Via Pietro Castellino 111, 80131 Naples, Italy

**Keywords:** myosin, athletes, movement asymmetry, sports, physical rehabilitation, genetics, Functional Movement Screen, exercise genomics

## Abstract

Asymmetry plays a major role in biology at all scales. This can be seen in the helix of DNA, the fact that the human heart is on the left side, or that most people use their right hand. A single protein such as Myosin 1D can induce helical motion in another molecule. This causes cells, organs, and even entire bodies to twist in a domino effect, causing left–right behaviour. More generally, athlete movements are often asymmetric and, during the physical rehabilitation after injury, the asymmetry is visually discernible. Herein, we review the molecular basis of the movement asymmetries and report on the available knowledge on the few therapeutics investigated so far such as meloxicam. From a more rehabilitative perspective, it is very important to use effective methods to control the process of resolving the injury-related movement asymmetry through the complex use of specialised exercises, measurements, and gait analysis, which can all provide useful information on the effectiveness of the rehabilitation plans. If for each athlete, the normal range of asymmetry is known, the asymmetry can be individually treated and the evolution can be monitored over time. Appropriate measures should be taken if the movement asymmetry is outside this range. In addition, genetic, physiological, and psychological factors relevant to athlete health should be considered in the process of assessing and improving exercise asymmetry, which we also discuss in this review. The main proposal of this work is that the movement asymmetries in athletes should be individually treated, while taking into account the athlete’s genetics, physical condition, and previous injuries.

## 1. Introduction

Asymmetry is one of the most important concepts in science in general and in biology in particular [1,2,3]. Movement asymmetries are correlated to molecular aspects, including the activity of particular proteins that determine the macroscopic behavioural asymmetric effects observed in organisms. This also holds true for the presence of asymmetries in sport [4,5]. Traditionally, sport science specialists have treated bilateral movement asymmetries in athletes as a sign of poor biomechanics and as a possible risk for injury [6,7,8,9,10]. This is true during injury rehabilitation when an athlete’s movements are restricted as a result of the injury [11,12,13]. However, the situation differs during exercise and training: there are many obviously asymmetric sports such as tennis, fencing, shot put, and javelin. Some sports are only partially asymmetric (e.g., high jump, long jump, football, and basketball), while some seem to be mostly symmetrical such as running and rowing [5]. It is also well known that asymmetries depend not only on the type of sport but on each individual and the applied test as well. Of course, asymmetric sports involve a certain amount of asymmetric movement. This leads to the uneven development of muscles on either side of an athlete’s body. If the asymmetries between the movements of the dominant and non-dominant limbs decrease, this can be a sign of fatigue in the dominant limbs or the presence of micro-injuries [14,15]. At the same time, the majority of experts conclude that sports performance, risk of injury, and the ability to adapt to exercise and recover post injury depend on such biological factors as genetic predisposition to injury, age, muscle elasticity, physical condition, and history of injury [16,17,18,19,20,21]. This suggests that the athlete’s biomechanical, kinematic, and kinetic parameters are tightly connected with the biomolecular processes in his or her muscles, ligaments, tendons, and bones. Without proper genetics, a good nutrition plan, and effective methods of bio-diagnostics, the athlete will fail to achieve good physical condition. Another aspect to consider is that there may also be perception-driven movement asymmetries caused by asymmetries in visual, audio, or other senses. For example, athletes whose attention is unevenly distributed in different directions are more likely to also have reduced reaction and movement asymmetries in certain directions, and as a result, will be more prone to injury [22,23]. In this paper, we explore the current knowledge on the molecular basis of the movement asymmetries and the role of movement asymmetries in sport in order to define the line between positive and negative aspects related to the asymmetries and find out how the biomechanical and biological processes in an athlete’s organism are connected to each other. The main applicative purpose of our work is to summarise modern strategies and methods adopted for the measurement, evaluation, and interpretation of movement asymmetries in sport and sports rehabilitation. This review also aims to understand the role of genetics in the formation and development of movement asymmetries in humans and especially in athletes.

## 2. Materials and Methods

As for the methodology used in this work, we explored the recent scientific literature relating to themes reported in the Section 1, as well as the keywords ‘movement asymmetry; sports; physical rehabilitation; genetics; nutrition’. We performed systematic literature research focused on biological aspects of movement asymmetries and implications regarding the control of movement asymmetries in athletes through the complex use of specialised exercises, measurements, and gait analysis. The research was conducted by searching PubMed with additional searches in Scopus, ScienceDirect, and Google Scholar databases. These databases were used to screen, collect, review, and analyse previous research information in order to compile the Section 3 and Section 4 of this review. All publications focusing on the criteria mentioned above were assessed. In our search, we excluded retracted publications and non-English articles. The emphasis was made on the recent publications published in the last 15 years containing valuable information about movement asymmetries and their biological and biomechanical aspects related to sports. The ages of participants were usually between 15 and 35 years.

**Figure 1:**
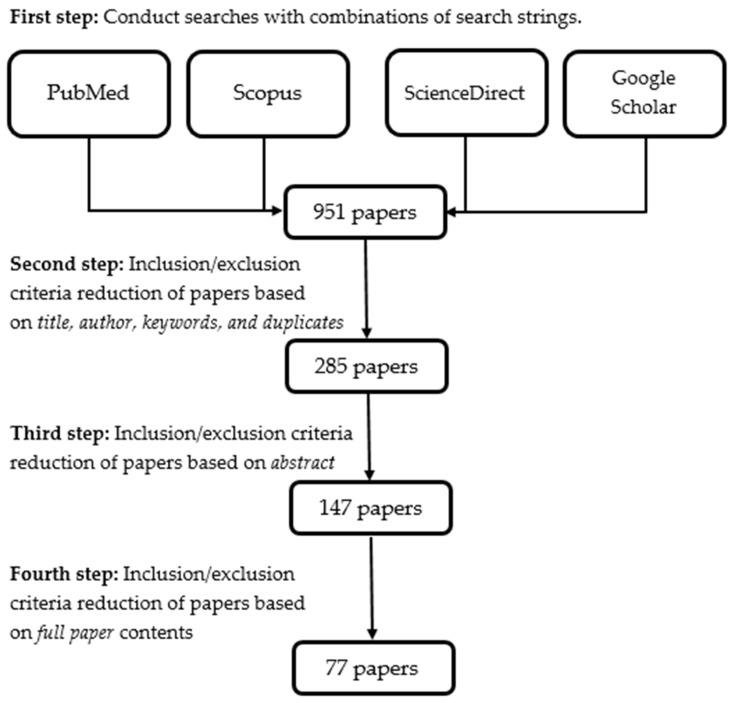
The process used for the selection of the papers examined and discussed in Section 3 and Section 4 of this review.

Out of 951 papers found at the first step of the process of paper selection, we selected 77 of the most relevant articles, and 874 papers have been excluded. Among the selected articles, 45 are dated between 2018 and 2023, 26 between 2012 and 2017, 5 between 2006 and 2011, and there is only one highly cited old article published in 1991. Almost all experimental studies have more than 15 subjects, and only three studies have the number of participants between 10 and 15. The process of the paper selection for Section 3 and Section 4 of the review is shown in Figure 1. The selected papers may be described as the optimal set of high-quality papers giving valuable information for understanding, measuring, and controlling movement asymmetries in athletes during training and rehabilitation. Of the 77 selected papers, 68 are indexed in PubMed or/and Scopus (54 in PubMed, 57 in Scopus). Of the rest of the 9 publications, 5 are indexed in Google Scholar, 2 in EBSCO, and 2 are international conference proceedings. The selected papers can be divided into the following categories: papers considering molecular aspects of movement asymmetries and sport genetics (18 papers), papers on the effect of movement asymmetries on sports performance and injury risk (16 papers), papers exploring the connection between reaction asymmetry and movement asymmetries (6 papers), papers dealing with movement asymmetry assessment methods and technologies (29 papers), and papers about the use of gait analysis for evaluation of movement asymmetries (8 papers).

## 3. Results

### 3.1. Assessing Movement Asymmetries Using Special Tests and Measurements

Specialists agree that asymmetry is relatively normal in sport since the vast majority of people have a certain degree of asymmetry within their physiognomy. Functional lower-limb asymmetries can be assessed using jumping tests including single-leg vertical jumps, countermovement jumps, and drop jumps [24,25,26,27,28,29,30]. Lower-limb strength asymmetries can be assessed using back squats, isometric squats, and isokinetic knee extensions [31,32,33,34]. According to large studies on the acceptable thresholds of asymmetry, 10–15% asymmetry is associated with abnormal differences between limbs [35,36]. Moreover, a ≤10% asymmetry has been suggested as a goal for athletes returning to sport [36]. At the same time, experts agree that coaches and physical rehabilitation specialists should treat movement asymmetries individually, taking into account an athlete’s genetic, physiological, and physical data [37,38,39,40]. It is very important to know the athlete’s optimal percentage of movement asymmetries related to the bilateral asymmetry characteristics of his/her sport. If the difference between the normal and observable asymmetries is too large (more than 10%) or if it is too small (for example, less than half of the normal asymmetry coefficient), then some measures should be taken to return to a normal level. In the first case, the difference should be normalised by improving the non-dominant side biomechanics, while in the second case, we should improve the dominant side biomechanics. Unfortunately, there is no general agreement on how to estimate the athlete’s movement asymmetries coefficient. For this purpose, we can use some standard exercises and measurements, for example, left and right-hand force measurements, single-hand exercises with balls and dumbbells, vertical jumps on a single leg, and ROMs (ranges of motion) measurements of active joints. Knowing the normal parameters for these exercises, coaches can detect abnormalities in an athlete’s performance and conduct a more detailed analysis of their condition.

### 3.2. Increasing the Accuracy of Estimation of Movement Asymmetries Using Motion Capture (MoCap), Force Plates, Treadmills and EMG

For a more accurate estimation of movement asymmetries, specialists use Motion Capture (MoCap) methods [41,42,43,44,45,46] and techniques for registering and measuring ground forces such as force plates and treadmills [47,48,49,50]. These methods allow us to estimate movement asymmetries during walking and running. Of course, the measurement results depend on the physical condition of the athlete—rested or tired, having muscle imbalance or dysfunction or not, etc. Importantly, asymmetries can lead to muscle imbalances, a decreased power output, and an increased risk of injury. In order to study an athlete’s muscle activity, specialists usually use electromyography (EMG), which is useful to help detect neuromuscular abnormalities [51,52,53]. Usually, movement asymmetries caused by an injury can be seen with the naked eye. In this case, an athletes’ movements may be constrained by effusion or pain leading to reduced muscle activation and weakness and can be easily detected without the use of special techniques [54]. We have a similar situation during an athlete’s rehabilitation after injury where compensation strategies can be observed in the injured limb and other areas of the body when compared with healthy controls [55]. In this case, an effective rehabilitation plan should be implemented by physical rehabilitation specialists, taking into account the character of the injury and the abnormalities observed [55].

### 3.3. Detection of Movement Asymmetries Using the Functional Movement Screen (FMS)

Not less important is the detection of movement asymmetries, associated with microtrauma that do not cause pain. In this case, an athlete can maintain a high level of performance by using compensatory movements and thereby increasing the risk of injury. For the estimation of movement asymmetries caused by a hidden microtrauma, specialists often use screening tools such as the Functional Movement Screen (FMS), which was identified to be the most popular field-based injury screening tool for identifying at-risk athletes. FMS consists of 7 basic exercises for detecting potential risks of injury. The main purpose of this screening tool is to determine whether the athlete is limited in fundamental movements [56,57]. Athletes with a microtrauma often demonstrate good performance during sporting events because they use compensatory movements to maintain a high level of performance. The use of FMS may help the coach or physical medicine specialist detect movement asymmetries during control exercises (Deep Squat, Hurdle Step, In-line Lunge, Active Straight-leg Raise, Trunk Stability Push-up, Rotary Stability, Shoulder Mobility). Only two exercises (Deep Squat and Trunk Stability Push-up) are symmetrical, while the other five include asymmetric movements and can reveal asymmetries in the movements of the athlete’s limbs. It has been shown that participating in multiple sports throughout multiple sporting seasons was associated with higher total FMS scores and fewer asymmetries, which may decrease subsequent risks of injury [58]. Some authors recommend that coaching/medical staff apply collective training programs with an emphasis on trunk stabilisation improvements (especially in younger players), while other specific deficits evidenced by FMS may be looked at on an individual basis or in sub-groups [59]. According to the work of Mokha et al., asymmetry or a low FMS individual test score was a better predictor of musculoskeletal injury than the composite FMS score [60]. Specialists also use FMS to obtain additional information about functional inter-limb asymmetries [61]. Australian scientists have validated FMS efficiency when combined with single-leg vertical jumps and previous injuries [61]. For a more detailed analysis, we can use the Motion Capture techniques: the parallel use of FMS and Motion Capture is recommended when the athlete has complex movement asymmetries that cannot be easily identified. Motion Capture allows us to measure flexion and extension angles more accurately [62,63,64]. In many cases, the use of two-dimensional programs is enough [65,66]. One such program is the 2D motion analysis Kinovea. It features tools to compare, slow down, measure, and annotate motions in videos. The methods described above may be used in different combinations or separately, but in all cases, one should take into consideration the athlete’s physical condition—rested or tired, pre-existing bilateral asymmetries, movement asymmetries related to their sport, and their history of injury. During rehabilitation and physical rehabilitation, specialists can use simple physical exercises and measurements to control the injured athlete’s condition. Alternatively, they can use the motion capture techniques to acquire more detailed information about the biomechanical parameters responsible for the athlete’s movement asymmetries. For each measurement, it is possible to calculate the corresponding movement asymmetries coefficient.

### 3.4. Biological Aspects of Movement Asymmetries

**Figure 2:**
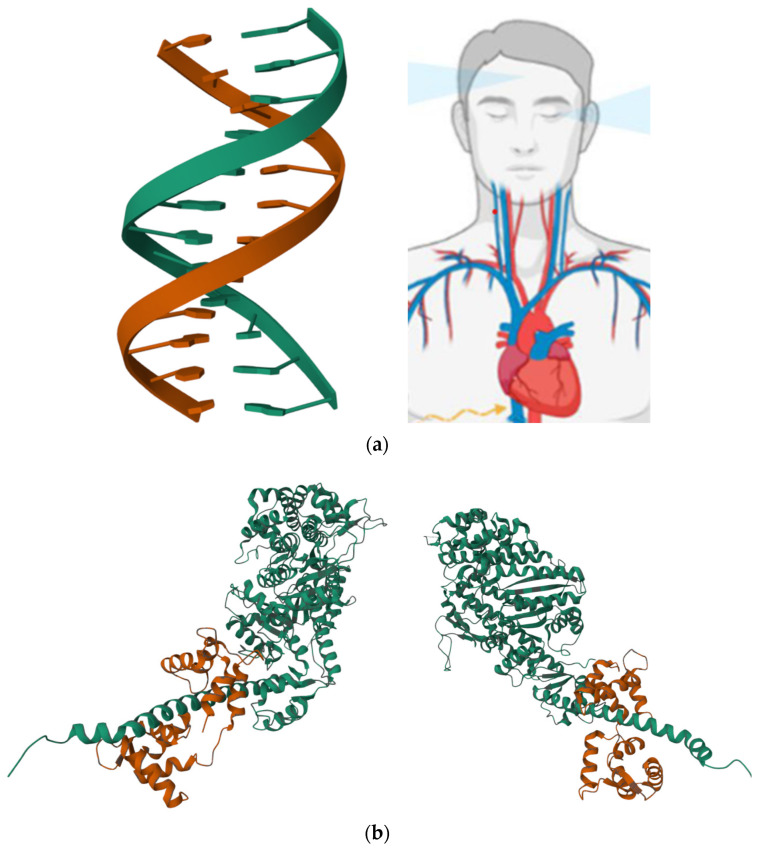
(**a**) Asymmetry of the double helix of DNA (the structure corresponds to the DNA dodecamer d(GGCAAAAAACGG)/d(CCGTTTTTTGCC) and is freely available at the link https://www.rcsb.org/3d-view/1FZX/0, accessed on 2 October 2023) and human body (adapted from https://www.mdpi.com/ijms/ijms-23-15150/article_deploy/html/images/ijms-23-15150-g001.png, accessed on 2 October 2023); (**b**) 3D views of the molecular structure of the human myosin I (the structure is freely available at the link https://www.rcsb.org/3d-view/4L79/1, accessed on 2 October 2023).

From the outside, most organisms appear symmetrical. However, many organs are asymmetrical such as the human heart that is shifted left of centre (Figure 2a), and overall, our world is fundamentally asymmetrical. An example is given by the double helix of DNA, the asymmetric division of stem cells, or the fact that as mentioned above, the human heart is positioned on the left (Figure 2a). However, the mechanism by which these asymmetries emerge and how they are linked to one another are aspects that still need to be elucidated. For example, whether macroscopic asymmetry is directly related to the chirality observed at the molecular level remains an open question. Working with Drosophila, Lebreton et al. found that a protein acting as the conserved molecular motor in the human body, such as myosin (Figure 2b), induces stereotypic chirality at all biological scales, from its F-actin transduction in vitro to the organ level, up to organismal behaviour [67]. Thus, this protein can produce de novo changes in shape and orientation not only at a nanometric but also macroscopic scale via chiral interactions with the actin cytoskeleton [67]. A molecular basis of lateral defects was also proven in humans [68]. In fact, an extremely rare novel missense variant in the MYO1D gene (Pro765Ser) was observed in correlation with visceral heterotaxy and left isomerism in polysplenia syndrome in humans. In more detail, the heterotaxy was found to result from the failure of the developing embryo to establish normal left–right asymmetry [68]. Remarkably, typical manifestations following the above-mentioned biomolecular alterations include an abnormal symmetry in the resulting organism. Surprisingly, a single mutated amino acid, Ser765, causes minor structural drifts and stability changes, which potentially affect the overall biophysical and functional properties of the resulting myosin including those connected with the microfilament motor activities. Importantly, MYO1D is ubiquitously expressed across several human tissues and is reported to induce severe phenotypes in knockout mouse models [68]. The above-described study constitutes the first report that a MYO1D genetic variant may cause left–right axis abnormalities in humans, adding to the already known evidence from zebrafish, frog, and Drosophila models [67]. Interestingly, the ability of Ser765 to induce small structural deviations and changes, altering the stability and the function of myosin, was proven using a bioinformatics-based multilevel structural analysis (structural modelling of protein variants, divergence, and stability). This study resulted in an important expansion of the previous knowledge on the genetic origin of human lateral defects, potentially opening the door to the identification of new drug targets and the development of personalised medicine for high-risk families [68].

#### Genetic Factors and Asymmetry

When dealing with movement asymmetries, we cannot ignore that, apart from the particular sport, the elasticity and quality of their muscles (density, separation, and definition), the quality of their nutritional plan and physical activity environments, as well as their history of previous injuries, such biological factors as the athlete’s DNA and, consequently, his/her genetic predisposition all play a crucial role. Genetics influences all areas of fitness and performance and has a large influence over strength, muscle size, and muscle fibre composition (fast or slow twitch), anaerobic threshold (AT), lung capacity, flexibility, and, to some extent, endurance. The following genetic factors should be given attention during training:

1. Genetic sensitivity to pain: according to the latest experimental data, a deficiency in the AMPD1 gene can cause symptoms such as pain and muscle weakness after exercise [16,17]. In asymmetric sports, dominant limbs are under more pressure than non-dominant limbs and, therefore, an athlete with higher pain tolerance will continue training with micro-injuries in the dominant limbs without feeling pain. This may lead to more serious injuries. In this case, the only way to detect abnormal changes in the athlete’s behaviour is to perform performance tests in time. If the dominant limbs move slower or have less force, then this may be a sign of a hidden injury. In the case of athletes with lower pain tolerance, even non-significant injuries will cause pain and impair movement performance. For such athletes, specialists recommend using yoga, biofeedback therapy, and exercises to increase pain tolerance.
2. Genetic predisposition to injuries: it was found that the genes AMPD1, GDF5, COL5A1, and IGF2 might be associated with increased injury occurrences in soccer players [16,18]. According to the latest research, the TT genotype of the GDF5 gene may be associated with an increased number of ankle and knee injuries as well as an increase in the total number of injuries and a decrease in the number of matches played [16]. This suggests that athletes who are genetically prone to certain types of injuries should be tested for such undetected injuries, especially in the dominant limbs.
3. Genetic need for increased recovery after a hard activity: the AMPD1 gene has been shown to be linked to such a need [17]. It was suggested that special attention should also be paid to the dominant limbs that will be under excessive physical load in asymmetric sports. The athlete should be given enough time for recovery. Obviously, the athletes with more endurance and a shorter recovery time will be favoured for such sports.
4. Genetic deficit of muscle elasticity or power: there is evidence suggesting that titin (encoded by the TTN gene), a protein that is expressed in cardiac and skeletal muscles, is one of the factors responsible for the muscles’ elasticity [19]. In some asymmetric sports, athletes with insufficient muscle elasticity may have issues with movements requiring a larger range of motion. Such athletes are recommended individual plans for training and nutrition [20,21].

In the next section, we will discuss the importance of assessing movement asymmetries in athletes through physical tests and during rehabilitation. It is obvious that the results of the tests depend on the athlete’s physical and psychological conditions. Tests may be taken after a period of rest in order to estimate whether the athlete is prepared for training and competition, or, alternatively, the athlete’s movement asymmetries can be tested after training in order to estimate how physical load and fatigue affect his physical condition and the bilateral asymmetries of his movements. Great relevance is also given to rehabilitation. If a non-dominant limb is injured, then the difference between non-dominant and dominant limbs will increase. Otherwise, if a dominant limb is injured, then the difference between the limbs may decrease in case of a minor injury, or the non-dominant limb may become even stronger in case of a more serious injury. In both cases, the injured limb should be trained with the purpose to regain its strength. The effectiveness of rehabilitation will depend on the athlete’s genetic ability to recover. Athletes with higher pain tolerance will recover faster than athletes with higher sensitivity to pain. The rehabilitation process can be hampered by poor nutrition or previous injuries.

## 4. Discussion

**Figure 3:**
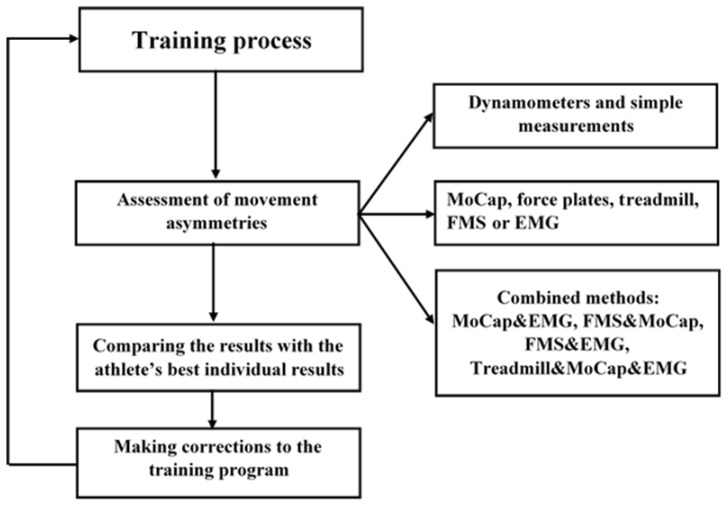
A schematic view of movement asymmetry assessment during training.

Knowledge of the above-mentioned biomechanical and biological aspects related to movement asymmetries can help us elaborate recommendations on how coaches and physical rehabilitation specialists should detect negative movement asymmetries and restore the normal state of the athlete. In this review, we consider two different types of negative movement asymmetries: (i) the first one is related to the negative movement asymmetries, which emerges during the training process and may be caused by factors such as improper training, overload, and poor nutrition. In this case, it is very important to detect such asymmetries in time and take the necessary measures to prevent their further development into more serious changes in the athlete’s organism; (ii) the second one is related to the post-injury negative movement asymmetries that are usually present during injury rehabilitation. In this case, physical rehabilitation specialists should provide an effective rehabilitation plan involving not only an optimal treatment plan but methods for evaluating its effectiveness as well. The detection of movement asymmetries of the first type will happen during the training process. During this stage, we suggest the following scheme consisting of three stages (see Figure 3). The first stage involves the estimation of the athlete’s basic physical and physiological parameters. Two procedures will be appropriate at this stage: (i) during exercises, coaches should keep control of the optimal parameters of the physical condition for each athlete. For example, force measurements using dynamometers give the best results for both hands, single-leg jumps (SLJ) give the best results for both legs, 100 m sprint results, and others; (ii) physical rehabilitation specialists should keep track of the athlete’s physiological parameters such as the heartbeat frequency, blood pressure, and blood oxygen saturation. Deterioration in the values of these parameters may be caused by fatigue. At this stage, we can detect slight negative changes in the athlete’s condition and make corrections to the training process.

The second stage aims to detect more hidden movement asymmetries and deviations in the athlete’s physical and physiological parameters. This stage includes the following procedures: (i) the use of more sophisticated techniques for the estimation of movement asymmetries such as motion capture, force plates and treadmills; (ii) the use of screening tools, such as FMS, which will help one predict possible trauma risks; and (iii) the assessment of the athlete’s muscle activity using EMG. EMG-based FMS can effectively detect early sports injuries and plays an effective role in reducing sports injuries. In the third stage, we can use different combinations of the above-mentioned methods, for example, MoCap and EMG, FMS and MoCap, MoCap with force plate and EMG, etc. This will increase the data integrity and consistency, making it more flexible and accurate.

**Figure 4:**
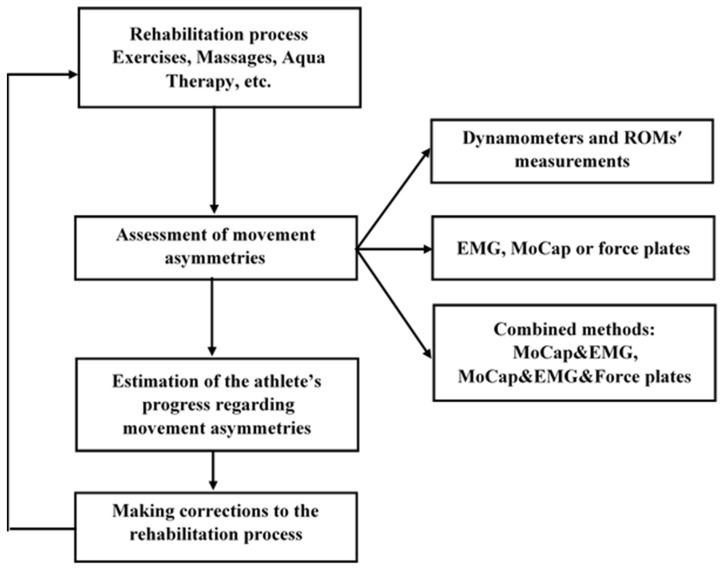
A schematic view of movement asymmetries assessment during rehabilitation.

During injury rehabilitation, the main task for physiotherapists is to normalise the condition of the injured joints and muscles, which, in most cases, restricts the athlete’s ability to move and manifests itself through movement asymmetries. For this purpose, specialists of physical rehabilitation use kinesiotherapy, massages, physiotherapy, and aqua therapy, among other methods (see Figure 4). The common practice for monitoring the progress of treatment is to use special control exercises, goniometers, gait analysis, and electromyography. These techniques allow one to estimate the muscle strength and range of movement (ROMs) of the injured muscles and joints. If the process of treatment does not bring noticeable changes to the athlete’s condition, the physiotherapist should modify the methods and techniques used during the treatment.

Alternatively, more sophisticated techniques for movement analysis should be considered. This can be a combination of different techniques such as MoCap, EMG, and force plates (see Figure 4). The parallel use of these methods makes it possible to understand the real connection between movement asymmetries and muscle activity. This will help the physiotherapist to find out which muscles are responsible for the athlete’s weakness and slow recovery. In some cases, injuries may be caused by asymmetries in the athlete’s reactions; they may have different reaction times in different directions or their attention may be mostly concentrated in certain directions over others. Such situations often occur in team sports, such as football or hockey, where the player has a certain position on the playing field that determines the focus of his attention. In sports, where attention is distributed unevenly in different directions, specialists recommend athletes pass special cognitive tests to check and improve their reaction time [22,23]. Another important factor determining an athlete’s susceptibility to injury may be the rules of the sport in question. According to recent works, sidedness has an impact on tactics and fighting techniques [69,70,71,72], similar to wrestling. For example, the study of the lateralization of injuries in judo is significant since determining the side of injury helps to identify the technical factors affecting the risk of injury [69]. Overall, several methods are commonly used for detecting and improving the negative movement asymmetries in athletes during training and rehabilitation. During training, in fact, it is important to identify the asymmetries in an athlete’s movements that may be caused by overload and micro-injuries. To accomplish this, there are a variety of methods at our disposal, including physical exercises and measurements to estimate muscle strength and joint range of motion. In addition, screening tools such as the Functional Movements Screen, Motion Capture technology, and electromyography can be used. The in-depth information captured by these tools will help us decrease the risk of injury and optimise the training process. During injury rehabilitation, an athlete’s movements are often asymmetrical where the asymmetries can be identified visually. In this case, it is important to use effective methods in order to control the process of elimination of the movement asymmetries related to the injury. The combined use of special exercises, measurements, and gait analysis may provide us with useful information about the effectiveness of the rehabilitation plan. We state that movement asymmetries should be treated individually, where each athlete will know his or her normal range of movement asymmetries. If movement asymmetries are out of this range, appropriate action must be taken. During the process of assessment and improvement of movement asymmetries, genetic, physiological, and psychological factors regarding the athlete’s health should also be taken into account. We have summarised the recommendations for controlling movement asymmetries in athletes in Table 1.

**Figure 5:**
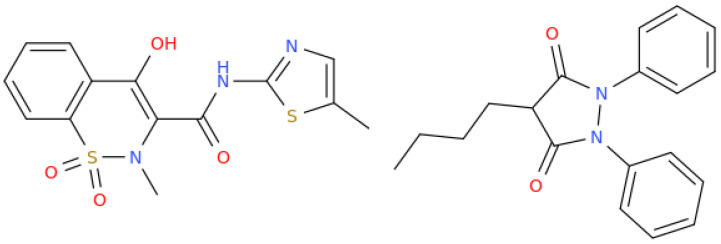
Structural representation of meloxicam (**left**) and phenylbutazone (**right**).

Few studies were conducted on therapeutics able to treat movement asymmetries. Meloxicam (use name for 4-hydroxy-2-methyl-N-(5-methyl-1,3-thiazol-2-yl)-1,1-dioxo-1lambda6,2-benzothiazine-3-carboxamide, Figure 5) tested together with other non-steroidal anti-inflammatory drugs such as phenylbutazone (4-butyl-1,2-diphenylpyrazolidine-3,5-dione, Figure 5) was shown to result in a reduction in head movement asymmetry [73]; however, the same activity was not observed in a subsequent study conducted on horses [74]. This latter finding seems to indicate that the movement asymmetries can be expressions of biological variation and are not necessarily related to pain/dysfunction that would be responsive to anti-inflammatory treatments.

## 5. Conclusions

Asymmetry is found at all levels in our world starting from chiral compounds to living organisms endowed with asymmetrical behaviours. From a biomolecular and mechanistic perspective, single residue mutations of motion-related proteins such as myosin can determine functional changes of the biological structures that lead to the macroscopic movement determining asymmetry as seen in insects and other organisms, including humans. Movement asymmetries are, at least to some extent, normal in humans and in particular in athletes. These could, anyway, show significant asymmetries in some cases that need to be carefully assessed and treated using physical therapy, which is the best weapon we can use to counteract the negative effects of asymmetries that, so far, lack any valid therapeutics to be used for the same purpose.

After a detailed analysis of the possible impacts of movement asymmetries on sports performance and an athlete’s physical condition, we can make the following conclusions. Movement asymmetries should be treated differently during training and rehabilitation. During training, knowing an athlete’s normal movement asymmetries will help coaches identify abnormal asymmetries in a timely manner and allow them to take appropriate actions. In asymmetric sports, special attention should be paid to the most active limbs that experience greater physical stress. Coaches can use simple control exercises and measurements to estimate the conditions of the dominant limbs of athletes. If the results of the testing are significantly worse than normal indicators, then we can look into the pathological changes in the athlete’s organism. During rehabilitation, physical rehabilitation specialists should put together an effective rehabilitation plan to eliminate movement asymmetries caused by injury. The progress of rehabilitation depends not only on the methods and technologies used but on genetic factors and previous injuries as well. In both cases, the effect of physical activity on the athlete’s movement asymmetries will depend on genetic factors such as sensitivity to pain, predisposition to injury, and injury recovery rate as well as muscle strength and elasticity. Other important factors include nutrition [75,76] and previous injuries. Health/fitness professionals are likely to face future challenges in how to optimise exercise training for individuals with desirable genes, as well as for motivating individuals without them to begin and/or continue exercising [77]. To meet these challenges, health/fitness professionals will need to become familiar with exercise genomics (i.e., studying how genetic and environmental factors affect exercise performance) and how it affects muscle strength, aerobic capacity, and other exercise-related phenotypes. Finally, we believe that all measures taken to counteract the movement asymmetries can lead to enhanced results when exercises are conducted in a green environment thanks to the huge benefits provided by nature, not only with therapeutic compounds [78,79,80], but also with forest bathing, river therapy, and other practices that can help improve human health status [81,82,83,84,85] and, more specifically, can have positive effects on treating movement asymmetries, that are important issues for athletes [86,87].

## Figures and Tables

**Table 1:** Recommendations for controlling main factors affecting movement asymmetries in athletes during training and rehabilitation.

Factors Affecting Movement Asymmetries	Recommendations	
	During Training	During Rehabilitation
Joints’ ranges of motion (ROM) asymmetry	Pay attention to the changes in the joints’ ranges of motion during training to keep them within optimal ranges.	Keep regular control of the joints’ ranges of motion (ROM) and asymmetries caused by the injury, and check their progress during rehabilitation.
Strength asymmetry	Keep control of the athlete’s inter-limb strength asymmetries during training to avoid overtraining.	Regularly check the athlete’s strength asymmetries related to the injury and estimate their improvement after the course of treatment.
Reaction asymmetry	Use cognitive tests and special exercises to avoid large differences between left and right limbs’ reaction times.	
Genetics	Take into account the athlete’s genetic predisposition to the particular sports activity.	Take into account the athlete’s genetic ability to recover and tolerate pain.
Nutrition	Elaborate an optimal nutrition plan taking into account the physical and physiological parameters of the athlete.	
The athlete’s physical and psychological condition	Create optimal training conditions for the athlete, including a good psychological environment.	Create a calm and friendly environment for the athlete undergoing rehabilitation.
Previous injuries history	Pay attention to the previous injuries suffered by the athlete and estimate their possible effect on his sports results.	Take into account the possible effect of the previous injuries on the athlete’s recovery rate.

## Data Availability

Not applicable.
